# Predictive value of the combined triglyceride-glucose and frailty index for cardiovascular disease and stroke in two prospective cohorts

**DOI:** 10.1186/s12933-025-02880-9

**Published:** 2025-08-04

**Authors:** Yi-Chang Zhao, Shi-Qi Wu, Jia-Kai Li, Zhi-Hua Sun, Bi-Kui Zhang, Rao Fu, Miao Yan

**Affiliations:** 1 Department of Pharmacy, The Second Xiangya Hospital, Central South University, Changsha, 410011 Hunan Province People’s Republic of China; 2 International Research Center for Precision Medicine, Transformative Technology and Software Services, Changsha, Hunan Province China; 3 National Clinical Research Center for Metabolic Diseases, Changsha, 410011 Hunan Province People’s Republic of China; 4 China Pharmaceutical University, Nanjing, 210009 Jiangsu Province People’s Republic of China; 5 Department of Clinical Pharmacy, The Second Xiangya Hospital of Central South University, Changsha, 410010 Hunan Province People’s Republic of China

**Keywords:** Triglyceride-glucose index, Frailty index, Cardiovascular disease, Stroke, Population-based cohort

## Abstract

**Background:**

The triglyceride-glucose (TyG) index is a validated surrogate for insulin resistance, while frailty reflects cumulative physiological decline. The combined impact of TyG-Frailty Index (TyGFI) has not been adequately explored. This study aimed to investigate the association between TyGFI and the risk of cardiovascular disease (CVD) and stroke.

**Methods:**

A total of 5448 participants from the China Health and Retirement Longitudinal Study (CHARLS) and 1139 participants from the U.S. National Health and Nutrition Examination Survey (NHANES) were included. Multivariable logistic regression models were used to estimate associations with CVD and stroke, adjusting for demographic, clinical, and lifestyle covariates. Restricted cubic spline (RCS) and subgroup analyses were employed to examine dose–response relationships and interaction effects.

**Results:**

Higher TyGFI levels were associated with older age, adverse metabolic parameters, and increased prevalence of hypertension, diabetes, and dyslipidemia. In fully adjusted models, the highest TyGFI quartile was significantly associated with increased risks of CVD (CHARLS: OR 15.09, 95% CI 9.65–23.60; NHANES: OR 4.98, 95% CI 2.04–12.19) and stroke (CHARLS: OR 21.12, 95% CI 6.44–69.23; NHANES: OR 12.98, 95% CI 2.58–65.17), with consistent dose–response trends confirmed by RCS analyses. Subgroup analyses further demonstrated the robustness of these associations across diverse demographic and clinical strata.

**Conclusions:**

TyGFI is a strong and independent predictor of CVD and stroke in two nationally representative cohorts. By integrating metabolic and functional risk dimensions, TyGFI provides a more comprehensive risk stratification tool, with significant implications for early identification and prevention of cardiovascular events in aging populations.

**Supplementary Information:**

The online version contains supplementary material available at 10.1186/s12933-025-02880-9.

## Introduction

Cardiovascular disease (CVD) and stroke remain the leading causes of mortality and disability worldwide, posing a substantial burden on global public health. Over 17 million people die each year from CVD, accounting for nearly one-third of all global deaths, with stroke ranking as the second leading cause of death and exhibiting a concerning shift toward younger populations [1, 2]. In China, stroke has become the leading cause of death and disability, with approximately 17.8 million prevalent cases, 3.4 million new cases annually, and 2.3 million stroke-related deaths reported in 2020 [3, 4]. As the aging population grows, the incidence and mortality rates of CVD and stroke continue to rise among older adults, underscoring the urgent need for more refined risk assessment tools to enable early prevention and intervention [5].

The triglyceride-glucose (TyG) index is calculated using routine biochemical parameters—fasting triglycerides and fasting plasma glucose—making it simple to apply in both clinical and epidemiological settings. It has been shown to correlate strongly with insulin resistance measured by the gold-standard euglycemic-hyper insulinemic clamp method. Guerrero-Romero et al. reported a significant inverse correlation between TyG and glucose disposal rate, with high diagnostic accuracy, supporting its utility as a simple and cost-effective alternative for assessing insulin sensitivity [6]. A growing body of literature has confirmed its predictive value for metabolic disorders and atherosclerotic diseases, including diabetes, coronary artery disease, heart failure, and stroke [7–10]. In addition to these primary outcomes, elevated TyG levels have also been associated with complex vascular outcomes such as carotid plaque progression, cardiovascular mortality, and post-stroke depression, expanding its utility in clinical risk stratification [11, 12]. In parallel, frailty index (FI)—a widely used and validated measure of biological aging—quantifies health status based on the accumulation of health deficits across multiple domains, including physical function, comorbidities, cognition, and psychological wellbeing. It reflects reduced physiological reserve and increased vulnerability to stressors, and is typically developed following the standardized 10-step procedure outlined by Theou et al., which ensures consistency in variable selection, coding, and index construction [13]. The FI has been independently linked to a range of adverse outcomes, including CVD, stroke, falls, cognitive decline, and all-cause mortality [14–16]. The high prevalence of frailty in older adults and its predictive relevance for poor health outcomes make it an essential component of geriatric risk models. Recent evidence has shown that worsening frailty is associated with increased CVD risk, whereas frailty remission may correspond with lower event rates [17].

Despite the established individual roles of TyG and frailty in predicting cardiovascular risk, they have largely been assessed in isolation. Few studies have explored their combined effect, even though metabolic dysfunction and physiological vulnerability frequently coexist in older adults. These coexisting conditions may act synergistically through mechanisms such as chronic inflammation, oxidative stress, and endothelial dysfunction to promote vascular damage and accelerate atherosclerosis [18, 19]. Moreover, traditional risk prediction models—such as the Framingham Risk Score—do not adequately incorporate aging-related variables such as frailty or low-grade inflammation, limiting their accuracy in elderly populations.

Therefore, this study aims to address an important gap by integrating the TyG index and FI into a unified risk construct that better reflects the dual burden of metabolic stress and biological aging. This approach is intended to improve cardiovascular risk stratification, particularly in older adults who are often underrepresented in traditional models. To address this gap, the present study introduces a novel composite risk indicator—the TyG-Frailty Index (TyGFI)—designed to capture the dual burden of metabolic stress and physiological vulnerability. Using data from two nationally representative cohorts, the China Health and Retirement Longitudinal Study (CHARLS) and the U.S. National Health and Nutrition Examination Survey (NHANES), we systematically examine the associations between TyGFI and the risks of CVD and stroke. Since the CHARLS cohort includes individuals aged 45 and above, participants under 45 were excluded from the NHANES sample to ensure cohort consistency and reduce age-related confounding. This threshold is supported by the China-PAR project, which defines elevated ASCVD risk beginning at age 45 for men and 55 for women. Accordingly, the TyGFI is designed for middle-aged and older adults (≥ 45 years), especially those with metabolic risk or early signs of functional decline, where traditional models may overlook aging-related factors [20]. Through multivariable logistic regression, restricted cubic spline (RCS) modeling, and stratified subgroup analyses, this study aims to evaluate the independent and joint predictive value of TyGFI. Our findings have the potential to inform a more nuanced and integrative approach to cardiovascular risk assessment, particularly among older adults with high multimorbidity burden.

## Methods

### Study design overview

**Fig. 1:**
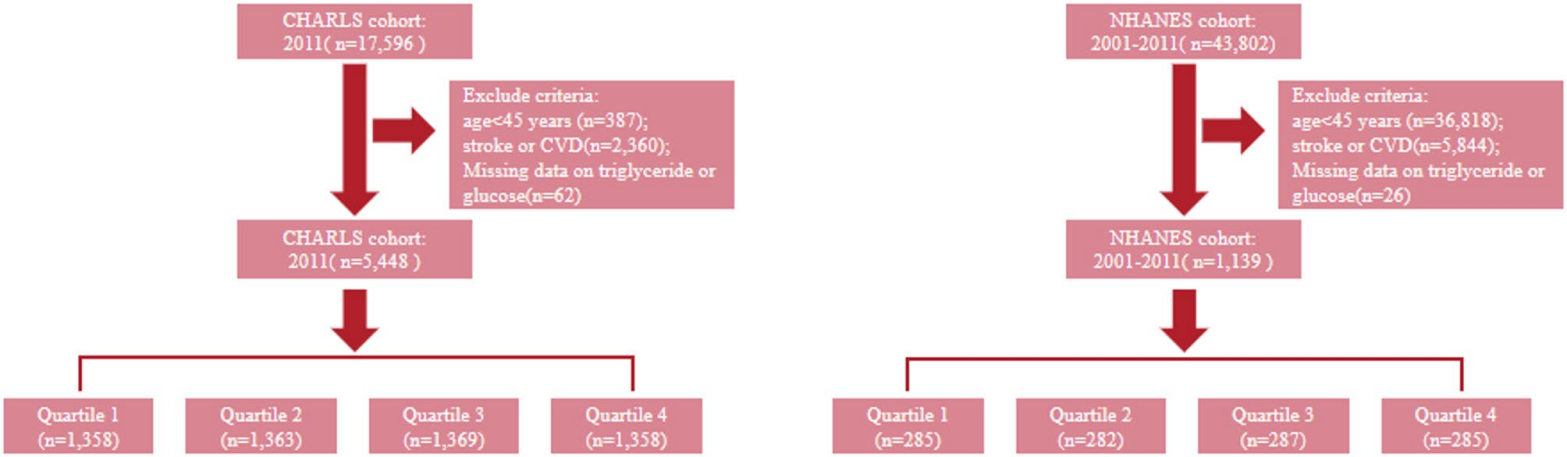
Flow diagram of participant selection from the CHARLS and NHANES cohorts. This figure illustrates the stepwise selection of study participants from the 2011 wave of the CHARLS and from the 2001–2011 cycles of the U.S. NHANES. Participants were excluded if they were younger than 45 years, had a history of stroke or CVD, or had missing values for serum triglyceride or glucose. The final eligible samples in each cohort were then divided into four groups (Quartiles 1–4) based on the distribution of the variable of interest (e.g., TyG index). The numbers shown under each box represent the sample size remaining at each stage of inclusion and exclusion

This study conducted a comprehensive analysis of the association between the combined TyG and FI and the incidence of CVD and stroke, adjusting for multiple potential confounders. Data were obtained from two nationally cohorts: the NHANES and the CHARLS. The findings were rigorously validated through sensitivity analyses. To ensure consistency and data quality across cohorts, we applied uniform inclusion and exclusion criteria. Participants were included if they were aged 45 years or older and had complete data on fasting triglycerides, fasting glucose, and variables required for constructing the FI. Participants with a history of stroke or CVD were excluded to minimize reverse causation. In the CHARLS cohort, baseline stroke and CVD status were determined through structured health interviews. Participants were excluded if they reported ever being diagnosed by a doctor with any major cardiovascular condition, including myocardial infarction, coronary heart disease, heart failure, or stroke, as recorded in the CHARLS health status module [21]. Similarly, in NHANES, CVD history was based on self-reported responses to the Medical Conditions Questionnaire (MCQ 160 series), which asks whether a doctor or health professional has ever diagnosed the participant with coronary heart disease, congestive heart failure, angina pectoris, heart attack, or stroke. Participants who responded “yes” to any of these items (MCQ160b–f) were classified as having pre-existing CVD [22]. We also excluded participants with missing values for triglycerides, fasting glucose, FI components, or outcome variables (CVD and stroke). Detailed procedures for data extraction from the two cohorts are depicted in the flowcharts (Fig. 1). The CHARLS cohort initially comprised 17,596 participants from the 2011 survey. The final analytical sample included 5,448 participants, stratified into four quartiles (n = 1358–1369). Meanwhile, the NHANES cohort yielded a final sample of 1139 participants, also divided into quartiles (n = 282–287).

### Two extensive observational cohort studies

NHANES Database Cohort: NHANES is a comprehensive health examination and nutritional assessment survey of adults and children conducted in the United States. The database includes demographic information, dietary habits, laboratory tests, physical examinations, and questionnaire responses collected between 2001 and 2011. The NHANES study protocol received approval from the Ethics Review Board of the National Center for Health Statistics, and written informed consent was obtained from all participants prior to data collection.

CHARLS Database Cohort: Data were also sourced from CHARLS, a nationally representative longitudinal survey targeting middle-aged and older adults aged 45 years and above in China, designed to capture health and economic dynamics within this population. CHARLS covers demographic information, physical health assessments, cognitive tests, biomarkers, and socioeconomic indicators. Ethical approval for CHARLS was provided by the Institutional Review Board at Peking University, and informed consent was obtained from all study participants.

### Data collection and definitions

The CHARLS dataset encompassed data collected between 2011 and 2015, while the NHANES dataset covered the period from 2001 to 2011, with baseline data specifically restricted to 2011 or earlier. Collected variables included demographic characteristics such as age, sex, and education level. Clinical measurements recorded included systolic blood pressure (SBP), diastolic blood pressure (DBP), body mass index (BMI), glucose, hemoglobin A1c (HbA1c), triglycerides (TG), total cholesterol (TC), high-density lipoprotein cholesterol (HDLC), and low-density lipoprotein cholesterol (LDLC). Lifestyle factors such as smoking and drinking status were documented, alongside medical histories of dyslipidemia, hypertension, and diabetes mellitus (DM). The TyG index was calculated using the formula: Ln [triglycerides (mg/dl) × glucose (mg/dl)/2] [6], which has been widely adopted in metabolic and cardiovascular research, and its validity has been reaffirmed by subsequent publications [7, 19]. In this study, the FI was constructed separately for the CHARLS and NHANES cohorts following the standardized deficit accumulation model proposed by Searle et al. [23]. To avoid circularity and ensure valid association analyses, cardiovascular disease–related items (e.g., stroke, heart failure) were excluded from both FI versions. In the CHARLS cohort, a 30-item FI (Supplementary file 1) was constructed following the deficit accumulation model, incorporating variables across core domains chronic conditions, physical functioning, depressive symptoms, and cognitive performance, in line with established protocols and publications [21, 24, 25]. In the NHANES cohort, a 49-item FI (Supplementary file 2) was adopted. leveraging a wider array of health-related indicators available in the U.S. dataset, covering comparable domains [22, 26]. Each variable was dichotomized or ordinally scaled and subsequently normalized to a 0–1 scale, with higher values indicating greater health deficits. The FI was calculated as the proportion of deficits present relative to the total number of items assessed. Due to differences in survey structure and item availability across CHARLS and NHANES, cross-cohort harmonization of the FI was not feasible. And cohort-specific FIs were constructed and analyzed separately to maintain internal consistency within each dataset. The combined TyG and FI was constructed using a multiplicative model: TyGFI = TyG × FI, as previously proposed in epidemiological analyses where product-based terms are used to reflect potential interaction effects between metabolic and functional deficits [27, 28].

### Weighting and representativeness

In the CHARLS cohort, a multistage, stratified sampling design was used, with probability proportional to population size and post-stratification at the provincial level, ensuring the representativeness of China’s middle-aged and older adult population. Unweighted analyses were conducted, following established practices in prior CHARLS studies [21]. For the NHANES cohort, all analyses were conducted in accordance with NHANES analytic guidelines, incorporating appropriate survey weights, stratification, and primary sampling unit variables. Specifically, the 2-year MEC examination weight WTMEC2YR was used as the probability weight, SDMVSTRA as the stratum variable, and SDMVPSU as the primary sampling unit. These design elements were incorporated to account for the complex, multistage, stratified, and clustered sampling scheme, ensuring that all reported estimates, standard errors, and 95% confidence intervals are representative of the non-institutionalized U.S. civilian population. All analyses utilized both interview and examination data to produce nationally representative estimates [22].

### Statistical analysis

Descriptive statistics were conducted to summarize the baseline demographic and clinical characteristics of the study population. Missing data were handled by excluding cases with missing values for variables required to construct the FI and outcome variables (CVD and Stroke). Only complete cases with no missing data for key variables were included in the analysis, with no imputation applied. Continuous variables were presented as means ± standard deviations (SD), and categorical variables as counts and percentages. Group comparisons were performed using one-way analysis of variance (ANOVA). Categorical variables were presented as frequencies and percentages, and compared using chi-square tests. All descriptive analyses were conducted separately for the CHARLS and NHANES cohorts using consistent statistical procedures. Logistic regression analyses were employed to evaluate associations between the combined TyGFI and outcomes CVD and stroke with adjustments for potential confounders. For covariate selection, we conducted a collinearity analysis and included only variables with a Variance Inflation Factor (VIF) less than 5. This selection process also referred to previously published literature (Supplementary file 4) [8, 19]. Both continuous TyGFI and categorical TyGFI (median-based quartiles) were analyzed, with odds ratios (ORs), 95% confidence intervals (CIs), and *p*-values reported. Restricted cubic spline (RCS) analysis was applied to evaluate nonlinear associations, visualized through plots indicating the probability of CVD and stroke. We used 3 knots to model the relationship between TyGFI and the outcomes (CVD and stroke). The selection of 3 knots was informed by established practices in the literature, providing a balance between model flexibility and avoiding overfitting [29, 30]. Stratified subgroup analyses were performed based on variables such as sex, smoking status, drinking status, hypertension, DM, dyslipidemia, and education. Results were visualized using forest plots. The study strictly adhered to STROBE guidelines and accounted for NHANES survey design and sampling weights. Post-hoc power analysis was conducted using G*Power 3.1 to assess whether the study sample size was sufficient to detect the observed associations. Calculations used ORs for TyGFI quartiles, event rates, and α = 0.05. Power ≥ 80% was considered adequate for identifying clinically meaningful associations.

## Result

### Baseline characteristics of the study population

According to the inclusion and exclusion criteria, a total of 5448 participants from the CHARLS cohort (female: 53.71%, mean age: 58.65 years) and 1139 from the NHANES cohort (female: 52.74%, mean age: 60.96 years) were included in the baseline analyses. Detailed participant characteristics are summarized in Table 1. Participants were categorized into quartiles (Q1–Q4) based on their TyGFI levels. Across both cohorts, individuals in higher TyGFI quartiles tended to be older and exhibited more adverse metabolic profiles. In CHARLS, average age increased from 56.4 in Q1 to 61.9 years in Q4, and in NHANES from 59.1 to 62.8 years. BMI followed a similar trend, rising from 23.2 to 23.9 kg/m^2^ in CHARLS and from 27.6 to 31.7 kg/m^2^ in NHANES. Metabolic biomarkers also showed significant changes. Fasting glucose increased from 104.7 to 116.7 mg/dL in CHARLS and from 100.9 to 128.2 mg/dL in NHANES. HbA1c rose from 5.1 to 5.4% in CHARLS and from 5.5 to 6.4% in NHANES. In both cohorts, TG, TC, and LDLC increased across TyGFI quartiles, while HDLC declined. Prevalence of chronic conditions also increased with TyGFI. In CHARLS, hypertension rose from 21% in Q1 to 56% in Q4, dyslipidemia from 34 to 46%, and DM from 8 to 22%. Similar increases were observed in NHANES, with DM rising from 25 to 64%. Smoking prevalence and lower educational attainment were more common in higher TyGFI groups. Notably, drinking frequency declined with TyGFI in CHARLS but showed no consistent pattern in NHANES.

**Table 1:** Baseline characteristics between CHARLS and NHANES cohorts

CHARLS cohort							NHANES cohort					
Variable	Total (n = 5448)	Q1 (n = 1358)	Q2 (n = 1363)	Q3 (n = 1369)	Q4 (n = 1358)	*p* value	Total (n = 1139)	Q1 (n = 285)	Q2 (n = 282)	Q3 (n = 287)	Q4 (n = 285)	*P* value
Sex						< 0.0001						0.27
Female	2926(53.71)	635(46.76)	704(51.65)	769(56.17)	818(60.24)		577(50.66)	143(50.18)	126(44.68)	150(52.26)	158(55.44)	
Male	2522(46.29)	723(53.24)	659(48.35)	600(43.83)	540(39.76)		562(49.34)	142(49.82)	156(55.32)	137(47.74)	127(44.56)	
Age	58.65 ± 8.75	56.37 ± 8.07	57.31 ± 8.35	58.99 ± 8.61	61.95 ± 8.92	< 0.0001	60.96 ± 0.35	59.08 ± 0.51	59.81 ± 0.58	62.87 ± 0.88	62.78 ± 0.79	0.002
Education						< 0.0001						< 0.001
High school or above	405( 7.43)	170(12.52)	99( 7.26)	91( 6.65)	45( 3.31)		302(26.51)	58(20.35)	60(21.28)	79(27.53)	105(36.84)	
Junior high school or below	2471(45.36)	656(48.31)	673(49.38)	612(44.70)	530(39.03)							
Illiterate	2572(47.21)	532(39.18)	591(43.36)	666(48.65)	783(57.66)		837(73.49)	227(79.65)	222(78.72)	208(72.47)	180(63.16)	
SBP	129.50 ± 21.07	124.75 ± 17.84	126.98 ± 19.69	131.17 ± 21.96	135.11 ± 22.93	< 0.0001	126.09 ± 0.93	123.27 ± 1.05	125.92 ± 1.93	128.06 ± 1.80	127.99 ± 1.34	0.05
DBP	75.44 ± 12.05	73.81 ± 10.93	74.87 ± 11.73	76.05 ± 12.55	77.05 ± 12.65	< 0.0001	70.78 ± 0.54	71.95 ± 0.65	72.68 ± 1.40	69.32 ± 1.37	68.53 ± 0.86	0.01
BMI	23.50 ± 3.76	23.22 ± 3.33	23.36 ± 3.63	23.53 ± 3.79	23.89 ± 4.19	< 0.0001	29.55 ± 0.45	27.61 ± 0.43	29.43 ± 0.75	30.03 ± 0.71	31.74 ± 0.80	0.001
Smoke						< 0.0001						0.002
No	3784(69.46)	881(64.87)	902(66.18)	985(71.95)	1016(74.82)		580(50.92)	164(57.54)	147(52.13)	134(46.69)	135(47.37)	
Yes	1664(30.54)	477(35.13)	461(33.82)	384(28.05)	342(25.18)		559(49.08)	121(42.46)	135(47.87)	153(53.31)	150(52.63)	
Drink						< 0.0001						0.43
No	3592(65.93)	801(58.98)	866(63.54)	940(68.66)	985(72.53)		194(17.03)	39(13.68)	48(17.02)	48(16.72)	59(20.70)	
Yes	1856(34.07)	557(41.02)	497(36.46)	429(31.34)	373(27.47)		945(82.97)	246(86.32)	234(82.98)	239(83.28)	226(79.30)	
Dyslipidemia						< 0.0001						0.002
No	3315(60.85)	902(66.42)	870(63.83)	807(58.95)	736(54.20)		203(17.82)	72(25.26)	60(21.28)	47(16.38)	24( 8.42)	
Yes	2133(39.15)	456(33.58)	493(36.17)	562(41.05)	622(45.80)		936(82.18)	213(74.74)	222(78.72)	240(83.62)	261(91.58)	
Hypertension						< 0.0001						< 0.0001
No	3421(62.79)	1071(78.87)	958(70.29)	789(57.63)	603(44.40)		443(38.89)	191(67.02)	118(41.84)	78(27.18)	56(19.65)	
Yes	2027(37.21)	287(21.13)	405(29.71)	580(42.37)	755(55.60)		696(61.11)	94(32.98)	164(58.16)	209(72.82)	229(80.35)	
DM						< 0.0001						< 0.0001
No	4721(86.66)	1250(92.05)	1235(90.61)	1178(86.05)	1058(77.91)		766(67.25)	252(88.42)	218(77.30)	179(62.37)	117(41.05)	
Yes	727(13.34)	108( 7.95)	128( 9.39)	191(13.95)	300(22.09)		373(32.75)	33(11.58)	64(22.70)	108(37.63)	168(58.95)	
Glucose	109.52 ± 34.52	104.65 ± 25.54	106.69 ± 27.35	110.11 ± 32.65	116.65 ± 47.15	< 0.0001	111.14 ± 2.11	100.91 ± 1.02	104.48 ± 1.48	114.94 ± 2.39	128.24 ± 4.98	< 0.0001
HbA1c	5.26 ± 0.79	5.12 ± 0.58	5.20 ± 0.68	5.27 ± 0.76	5.43 ± 1.03	< 0.0001	5.89 ± 0.07	5.52 ± 0.03	5.61 ± 0.04	6.14 ± 0.10	6.44 ± 0.13	< 0.0001
TG	130.89 ± 95.81	119.67 ± 84.38	122.39 ± 83.35	137.27 ± 103.92	144.21 ± 106.99	< 0.0001	129.06 ± 4.50	110.05 ± 4.75	123.93 ± 6.98	128.76 ± 5.13	159.95 ± 8.23	< 0.001
TC	193.39 ± 37.54	189.31 ± 37.24	192.10 ± 36.16	194.67 ± 37.38	197.48 ± 38.88	< 0.0001	129.06 ± 4.50	110.05 ± 4.75	123.93 ± 6.98	128.76 ± 5.13	159.95 ± 8.23	< 0.001
HDLC	51.43 ± 15.25	52.03 ± 15.06	51.93 ± 15.12	51.23 ± 14.95	50.51 ± 15.83	0.03	54.59 ± 1.08	57.93 ± 1.11	55.17 ± 1.20	54.77 ± 1.49	49.40 ± 1.67	< 0.001
LDLC	116.21 ± 34.64	113.87 ± 33.73	116.46 ± 33.28	116.47 ± 35.49	118.04 ± 35.89	0.02	117.88 ± 1.35	122.28 ± 2.32	118.75 ± 2.98	115.91 ± 2.70	113.15 ± 2.79	0.06
TyG	4.68 ± 0.33	4.63 ± 0.31	4.65 ± 0.31	4.71 ± 0.32	4.75 ± 0.35	< 0.0001	4.71 ± 0.02	4.61 ± 0.02	4.67 ± 0.03	4.74 ± 0.02	4.88 ± 0.03	< 0.0001
FI	0.09 ± 0.06	0.03 ± 0.02	0.07 ± 0.03	0.10 ± 0.03	0.16 ± 0.07	< 0.0001	0.16 ± 0.01	0.07 ± 0.00	0.12 ± 0.00	0.18 ± 0.00	0.30 ± 0.00	< 0.0001
TyGFI	0.46 ± 0.30	0.16 ± 0.06	0.33 ± 0.04	0.49 ± 0.05	0.87 ± 0.29	< 0.0001	0.76 ± 0.03	0.30 ± 0.01	0.57 ± 0.01	0.86 ± 0.01	1.46 ± 0.03	< 0.0001

This table presents key demographic, clinical, lifestyle, and biochemical variables used in the analysis. SBP, systolic blood pressure; DBP, diastolic blood pressure: Measured in mmHg; BMI, body mass index: Defined as weight (kg) divided by height squared (m^2^); Smoking status: *yes* (current/former smoker) or *no* (never smoked); Drinking status: *yes* (current/former drinker) or *no* (never consumed alcohol); Dyslipidemia, hypertension, and diabetes (DM): binary variables indicating the presence (*yes*) or absence (*no*) of these conditions; Glucose: fasting glucose (mg/dL); HbA1c: glycated hemoglobin (%), indicating longterm glucose control; TG, fasting triglycerides (mg/dL); TC, total cholesterol; HDLC, highdensity lipoprotein cholesterol; LDLC, lowdensity lipoprotein cholesterol): Blood lipid levels (mg/dL); TyG, triglyceride glucose index: A metabolic risk marker calculated as: $TyG=\text{log}\left(\text{Fasting } \, \text{triglycerides}\times \text{Fasting } \, \text{Glucose}\right)/2$; FI, frailty index: indicator of frailty status; TyGFI, TyGFrailty index

### Association between TyGFI index and cardiovascular disease risk

The association between the TyGFI and the risk of CVD was evaluated in both the CHARLS and NHANES cohorts using logistic regression models with increasing levels of covariate adjustment (Table 2). None of the variables showed multicollinearity (Supplemental file 4). In the CHARLS cohort, a strong and graded association was observed between TyGFI quartiles and CVD risk. Compared to participants in the lowest quartile (Q1), those in Q2, Q3, and Q4 showed significantly elevated odds of cardiovascular events. In the fully adjusted model (Model 3), the odds ratios (ORs) for CVD were 3.55 (95% CI 2.21–5.70) in Q2, 7.77 (95% CI 4.96–12.18) in Q3, and 15.09 (95% CI 9.65–23.60) in Q4 (*p* for trend < 0.0001), which were consistent across all models. In the NHANES cohort, similar trends were observed, though with slightly attenuated effect sizes. In Model 3, compared with Q2 (reference), Q3 had an OR of 3.41 (95% CI 1.36–8.56) and Q4 had an OR of 4.98 (95% CI 2.04–12.19), both statistically significant. The *p* for trend remained highly significant (*p* < 0.001) in all models except Model 2 (*p* = 0.11), which still showed elevated point estimates. When TyGFI was analyzed as a continuous variable using its median value, the association with CVD remained robust across all models in both cohorts. After adjusting for age, sex, lifestyle factors, blood pressure, glycemic and lipid parameters, and socioeconomic variables (Model 3), TyGFI remained independently associated with increased cardiovascular risk. These findings from two nationally representative cohorts underscore a consistent and independent relationship between higher TyGFI and elevated risk of CVD, reinforcing the potential clinical utility of TyGFI as a composite marker for cardiovascular risk stratification as also shown in Table 2.

**Table 2:** Association between TyGFrailty Index and cardiovascular events risk in CHARLS and NHANES cohorts

Character	Crude model		Model 1		Model 2		Model 3	
	95%CI	*P*	95%CI	*P*	95%CI	*P*	95%CI	*P*
CHARLS cohort								
CVD ~ TyGFI	7.29(5.71, 9.31)	< 0.0001	7.28(5.62, 9.43)	< 0.0001	6.93(5.31, 9.05)	< 0.0001	7.08(5.39, 9.30)	< 0.0001
CVD ~ TyGFI(Q1–Q4)								
Q1	ref		ref		ref		ref	
Q2	3.48(2.17, 5.58)	< 0.0001	3.48(2.17, 5.57)	< 0.0001	3.43(2.14, 5.50)	< 0.0001	3.55(2.21, 5.70)	< 0.0001
Q3	7.68(4.93, 11.97)	< 0.0001	7.67(4.92, 11.98)	< 0.0001	7.42(4.75, 11.60)	< 0.0001	7.77(4.96, 12.18)	< 0.0001
Q4	14.82(9.61, 22.85)	< 0.0001	14.79(9.55, 22.93)	< 0.0001	14.1(9.07, 21.92)	< 0.0001	15.09(9.65, 23.60)	< 0.0001
p for trend(character2integer)		< 0.0001		< 0.0001		< 0.0001		< 0.0001
NHANES cohort								
CVD ~ TyGFI	5.81(3.76, 8.99)	< 0.0001	6.41(3.97, 10.34)	< 0.0001	6.67(2.63, 16.92)	0.01	6.77(3.41, 13.44)	< 0.0001
CVD ~ TyGFI(Q1-Q4)								
Q1	ref		ref		ref		ref	
Q2	4.56(1.54, 13.53)	0.01	4.19(1.45, 12.14)	0.01	3.76(1.41, 10.07)	0.22	3.41(1.36, 8.56)	0.01
Q3	7.20(2.77, 18.70)	< 0.001	5.96(2.24, 15.85)	0.002	5.66(2.31, 13.85)	0.15	4.98(2.04, 12.19)	0.001
Q4	15.06(6.18, 36.72)	< 0.0001	15.17(5.77, 39.83)	< 0.0001	13.70(5.02, 37.37)	0.11	11.74(4.37, 31.58)	< 0.0001
*p* for trend(character2integer)		< 0.0001		< 0.0001		0.01		< 0.001
TyGFI (Q1–Q4)								
crudel model: TyGFI (Q1–Q4)								
Model 1: TyGFI (Q1–Q4), Age, Sex								
Model 2: TyGFI (Q1–Q4), Age, Sex, Smoke, Drink, SBP, DBP, HbA1c, TC, HDLC, LDLC								
Model 3: TyGFI (Q1–Q4), Age, Sex, Smoke, Drink, SBP, DBP, HbA1c, TC, HDLC, LDLC,Education, Hypertension, DM, Dyslipidemia, Marital status, BMI								

This table presents the association between the TyGFrailty Index (TyGFI) and CVD events in the CHARLS and NHANES cohorts. Results are expressed as odds ratios (ORs) with 95% confidence intervals (CIs) across quartiles (Q1–Q4) of TyGFI, using Q1 as the reference group

Model Adjustments:

Crude: Unadjusted, including only TyGFI Qs

Model 1: Adjusted for age and sex

Model 2: Further adjusted for Smoke, Drink, SBP, DBP, HbA1c, TC, HDLC, LDLC

Model 3: Fully adjusted, additionally including Education, Hypertension, DM, Dyslipidemia, Marital status, BMI

The ORs represent the relative odds of CVD events for individuals in Q2–Q4 compared to Q1. An OR > 1 indicates an increased risk, while an OR < 1 indicates a reduced risk. Statistically significant associations (*p* < 0.05) are highlighted in the table. The *p*-value for trend assesses the significance of the overall association across TyGFI Qs

### Subgroup analysis for TyGFI index and cardiovascular disease risk

**Fig. 2:**
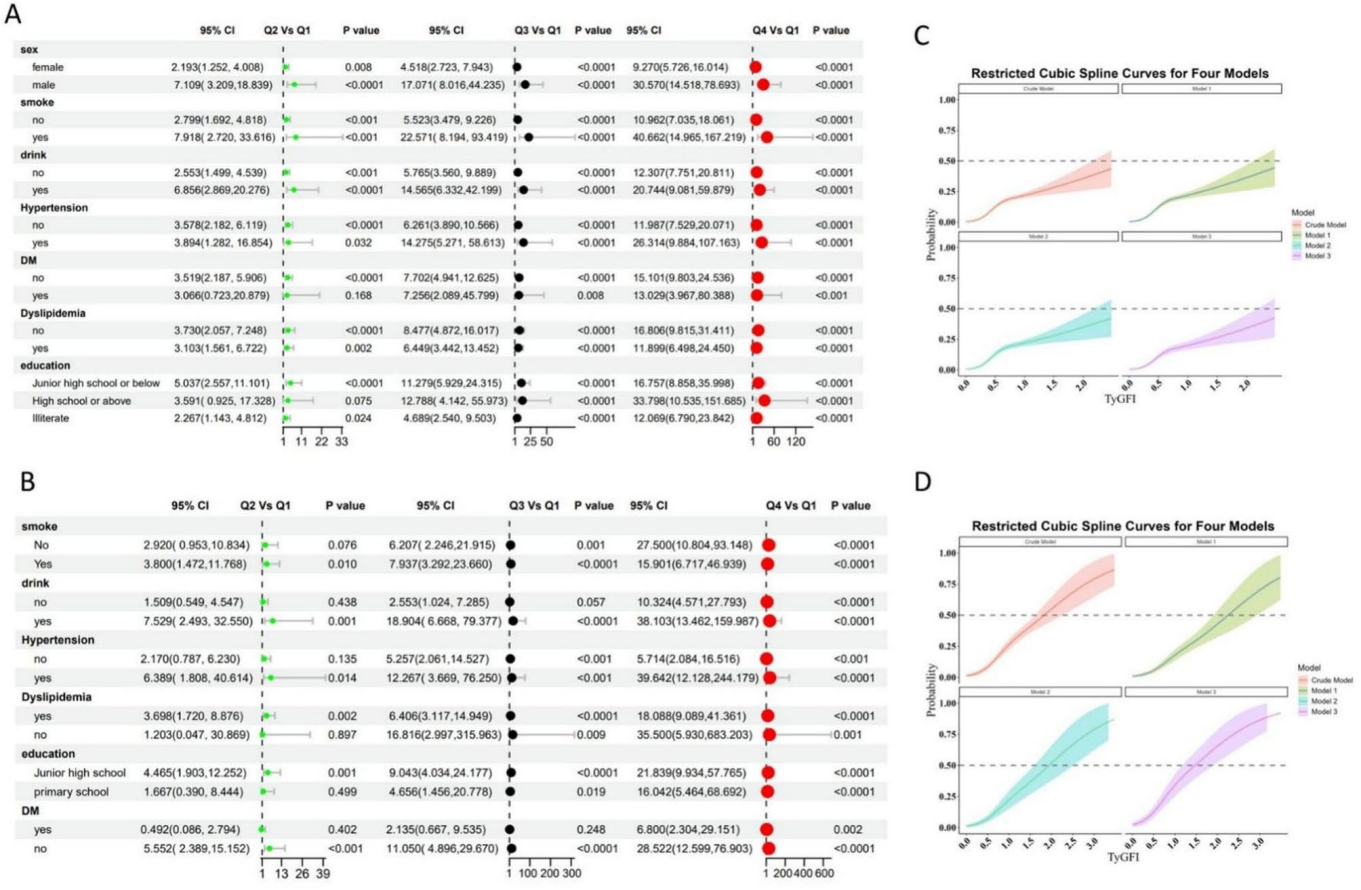
Multivariable logistic regression results and restricted cubic spline curves of the association between TyG-FI and CVD risk in the CHARLS and NHANES cohorts. **A** A forest plot from the CHARLS cohort showing the results of multivariable logistic regression analyses for CVD as the outcome. It displays the adjusted OR, 95% CI, and *p*-values across four different models. **B** A forest plot from the NHANES cohort showing the results of multivariable logistic regression analyses for CVD as the outcome. It similarly displays the OR, 95% CI, and *p*-values across four different models. **C** A dose–response curve from the CHARLS cohort based on RCS analysis, illustrating the relationship between TyG-FI and CVD risk. The shaded area represents the 95% confidence interval. **D** A dose–response curve from the NHANES cohort based on RCS analysis, illustrating the relationship between TyG-FI and CVD risk. The shaded area represents the 95% confidence interval

Subgroup analyses were conducted for both the CHARLS and NHANES cohorts, focusing on the association between TyGFI and CVD risk. The results from the CHARLS cohort (Fig. 2A) demonstrated a significant and consistent increase in CVD risk across TyGFI quartiles, with stronger associations observed among males, smokers, drinkers, and individuals with hypertension, diabetes mellitus (DM), and dyslipidemia. Participants with lower education levels also exhibited higher risk estimates. The RCS analysis (Fig. 2C) indicated a nonlinear positive association between TyGFI and CVD risk, further reinforcing the findings from the categorical quartile analysis.

Similarly, in the NHANES cohort (Fig. 2B), a stepwise increase in CVD risk was observed with higher TyGFI quartiles. The subgroup analysis revealed particularly strong associations among smokers, drinkers, and individuals with hypertension, DM, and dyslipidemia. Educational disparities were also noted, with lower education levels correlating with elevated CVD risk. RCS analysis (Fig. 2D) confirmed the nonlinear relationship between TyGFI and CVD, demonstrating a consistent trend across different adjustment models.

### Association between TyGFI index and stroke risk

Table 3 presents the associations between the TyGFI and stroke across the CHARLS and NHANES cohorts. In both cohorts, higher TyGFI levels were significantly associated with an increased risk of stroke. In the CHARLS cohort, each unit increase in TyGFI was associated with a markedly elevated risk of stroke across all models. In the fully adjusted Model 3, the odds ratio (OR) was 8.99 (95% CI 5.53–14.62, *p* < 0.0001). When TyGFI was analyzed by quartiles, a clear dose–response relationship was observed. Compared to the reference group (Q1), individuals in Q3 and Q4 had significantly higher risks of stroke in all models. In Model 3, the OR were 8.22 (95% CI 2.45–27.58, *p* < 0.001) for Q3 and 21.12 (95% CI 6.44–69.23, *p* < 0.0001) for Q4, with a significant trend across quartiles (*p* for trend < 0.0001). Similar patterns were observed in the NHANES cohort. Although the associations were somewhat attenuated compared to CHARLS, the trend remained significant. In the fully adjusted Model 3, the OR for Q3 and Q4 were 6.36 (95% CI 1.44–28.00, *p* = 0.02) and 12.98 (95% CI 2.58–65.17, *p* = 0.004), respectively, with a consistent trend across quartiles (*p* for trend < 0.001). The OR for Q2 in both cohorts did not reach statistical significance.

**Table 3:** Association Between TyGFrailty Index and stroke risk in CHARLS and NHANES Cohorts

Character	Crude model		Model 1		Model 2		Model 3	
	95%CI	P	95%CI	P	95%CI	P	95%CI	P
CHARLS cohort								
g ~ ~ stroke ~ TyGFI	7.67(5.03, 11.67)	< 0.0001	8.61(5.50, 13.48)	< 0.0001	8.78(5.46, 14.13)	< 0.0001	8.99(5.53, 14.62)	< 0.0001
stroke ~ TyGFI(Q1–Q4)								
Q1	Ref		Ref		Ref		Ref	
Q2	3(0.81, 11.12)	0.10	3.09(0.83, 11.44)	0.09	3.04(0.82, 11.27)	0.10	3.04(0.82, 11.29)	0.10
Q3	8.06(2.42, 26.83)	< 0.001	8.55(2.56, 28.52)	< 0.001	8.17(2.44, 27.32)	< 0.001	8.22(2.45, 27.58)	< 0.001
Q4	19.44(6.07, 62.26)	< 0.0001	21.41(6.62, 69.24)	< 0.0001	20.54(6.31, 66.83)	< 0.0001	21.12(6.44, 69.23)	< 0.0001
p for trend(character2integer)		< 0.0001		< 0.0001		< 0.0001		< 0.0001
NHANES cohort								
Stroke ~ TyGFI	5.46(2.98, 9.99)	< 0.0001	5.16(3.03, 8.81)	< 0.0001	5.89(2.44, 14.22)	0.01	6.13(3.58, 10.51)	< 0.0001
Stroke ~ TyGFI(Q1–Q4)								
Q1	Ref		Ref		Ref		Ref	
Q2	4.30(0.65, 28.40)	0.12	3.93(0.63, 24.56)	0.13	3.75(0.64, 21.80)	0.36	4.17(0.72, 24.24)	0.11
Q3	7.56(1.58, 36.12)	0.01	6.08(1.25, 29.63)	0.03	6.24(1.32, 29.54)	0.24	6.36(1.44, 28.00)	0.02
Q4	15.38(3.32, 71.31)	0.002	13.12(2.75, 62.54)	0.004	12.91(2.36, 70.67)	0.19	12.98(2.58, 65.17)	0.004
*p* for trend(character2integer)		< 0.001		< 0.001		0.03		< 0.001
TyGFI (Q1–Q4)								
crudel model: TyGFI (Q1–Q4)								
model 1: TyGFI (Q1–Q4), Age, Sex								
model 2: TyGFI (Q1–Q4), Age, Sex, Smoke, Drink, SBP, DBP, HbA1c, TC, HDLC, LDLC								
model 3: TyGFI (Q1–Q4), Age, Sex, Smoke, Drink, SBP, DBP, HbA1c, TC, HDLC, LDLC, Education, Hypertension, DM, Dyslipidemia, Marital status, BMI								

This table presents the association between the TyG Frailty Index (TyGFI) and stroke events in the CHARLS and NHANES cohorts. The results are expressed as OR with 95% CI across quartiles (Q1–Q4) of TyGFI, using Q1 as the reference group

Model Adjustments:

Crude: Unadjusted, including only TyGFI quartiles;

M1: Adjusted for age and sex;

M2: Further adjusted for Smoke, Drink, SBP, DBP, HbA1c, TC, HDLC, LDLC;

M3: Fully adjusted, additionally including Education, Hypertension, DM, Dyslipidemia, Marital status, BMI

The ORs represent the relative risk of stroke for individuals in Q2–Q4 compared to Q1. An OR > 1 suggests an increased risk, while an OR < 1 suggests a reduced risk. Statistically significant associations (*p* < 0.05) are highlighted in the table. The *p* trend assesses the overall association across TyGFI quartiles

### Subgroup analysis for TyGFI index and stroke risk

**Fig. 3:**
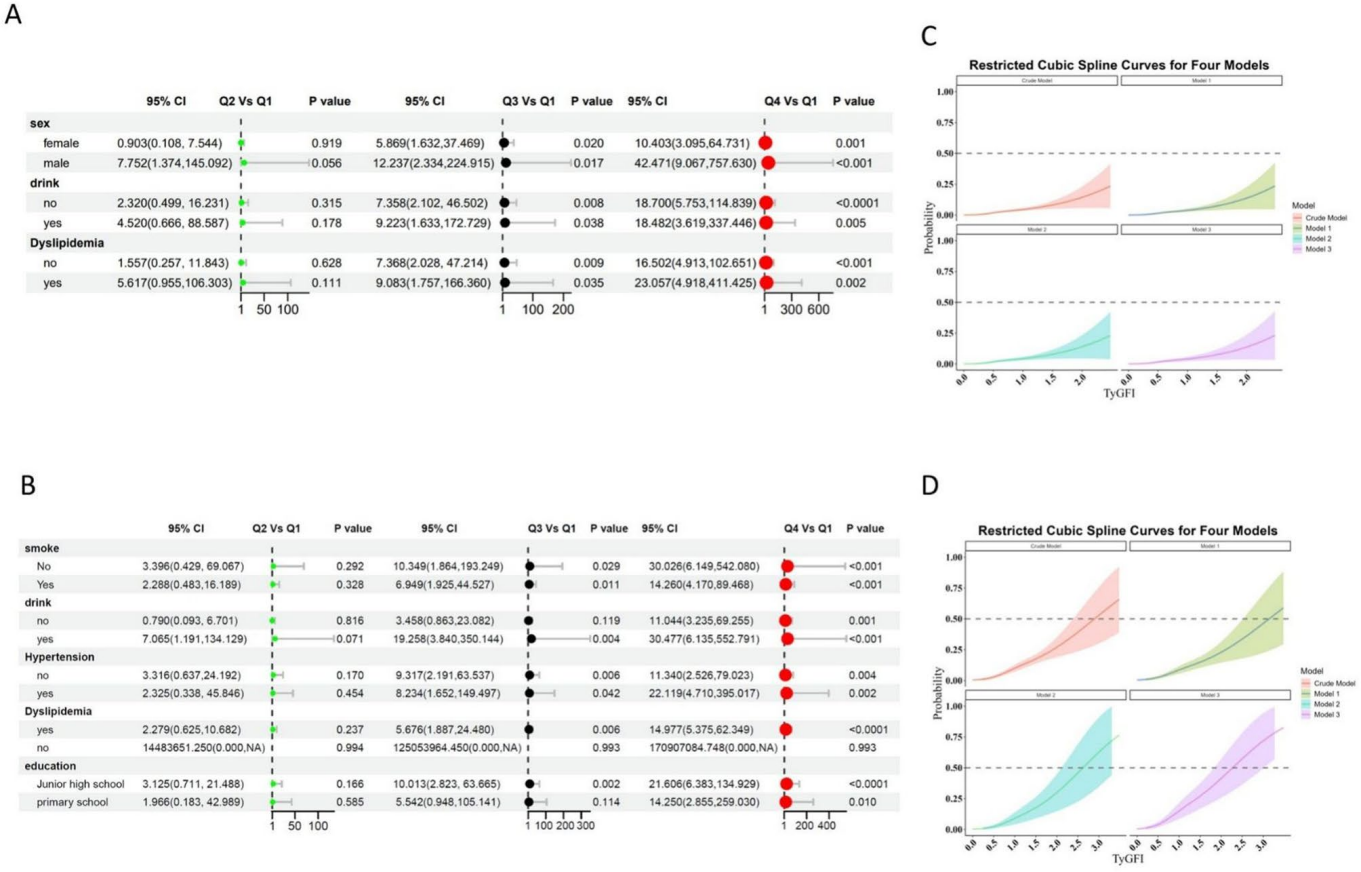
Multivariable logistic regression results and restricted cubic spline curves of the association between TyG-FI and stroke risk in the CHARLS and NHANES cohorts. **A** A forest plot from the CHARLS cohort showing the results of multivariable logistic regression analyses for stroke as the outcome. It displays the adjusted OR, 95% CI, and* p*-values across four different models. **B** A forest plot from the NHANES cohort showing the results of multivariable logistic regression analyses for stroke as the outcome. It similarly displays the OR, 95% CI, and *p*-values across four different models. **C** A dose–response curve from the CHARLS cohort based on RCS analysis, illustrating the relationship between TyG-FI and stroke risk. The shaded area represents the 95% confidence interval. **D** A dose–response curve from the NHANES cohort based on RCS analysis, illustrating the relationship between TyG-FI and stroke risk. The shaded area represents the 95% CI

Subgroup analyses were also performed to assess the relationship between TyGFI and stroke risk across different demographic and clinical subgroups within the CHARLS and NHANES cohorts. In the CHARLS cohort (Fig. 3A), a clear trend emerged, showing a significant escalation in stroke risk as TyGFI quartiles increased. Compared with participants in the lowest quartile, those in Q4 exhibited the highest stroke risk, particularly among males, individuals who consumed alcohol, and those diagnosed with dyslipidemia. Males in Q4 had markedly greater odds of stroke (OR 42.47, 95% CI 9.067–157.63, *p* < 0.001), as did drinkers (OR 18.48, 95% CI 3.619–347.36, *p* = 0.005) and participants with dyslipidemia (OR 23.06, 95% CI 4.918–411.43, *p* = 0.002). The RCS analysis (Fig. 3C) further illustrated a nonlinear association, demonstrating an accelerating increase in stroke probability at higher TyGFI levels.

Findings from the NHANES cohort (Fig. 3B) were consistent, revealing a stepwise elevation in stroke risk with increasing TyGFI quartiles. The association was particularly pronounced among those with hypertension, dyslipidemia, and lower educational attainment. Participants with hypertension in Q4 experienced a significantly heightened risk (OR 11.34, 95% CI 2.526–79.023, *p* = 0.004), as did those with dyslipidemia (OR 14.98, 95% CI 5.375–62.349, *p* < 0.001). Moreover, individuals with only primary school education demonstrated a stronger association with stroke risk (OR 14.25, 95% CI 0.855–259.03, *p* = 0.010). The RCS analysis (Fig. 3D) corroborated these findings, confirming a nonlinear dose–response relationship between TyGFI and stroke risk, with a sharp increase in probability observed at higher TyGFI values.

### Post-hoc power analysis

Estimated event rates were based on previously reported prevalence values and sample sizes. In CHARLS (n = 5448), the estimated power exceeded 99% for detecting ORs of 15.09 for CVD and 21.12 for stroke. In NHANES (n = 1139), the power was approximately 93% for detecting a CVD OR of 4.98 and 80% for a stroke OR of 12.98. These findings indicate that the study had sufficient statistical power (≥ 80%) to detect clinically meaningful associations, even with relatively low stroke event rates in NHANES. Full details of parameters and results are presented in Supplementary file 3.

### Sensitivity analyses

To assess the robustness of our findings and address potential biases, we conducted two sets of sensitivity analyses: one evaluating sample selection bias and the other assessing the impact of extreme values. Baseline comparisons between included and excluded participants showed that in CHARLS, the excluded group (1.7%) differed significantly on several variables, including age, sex, blood pressure, HDL-C, and chronic disease prevalence (Supplementary file 5). However, the small proportion limits the likelihood of significant selection bias. In NHANES, the excluded group (1.1%) showed no major differences apart from slightly higher rates of hypertension and diabetes (Supplementary file 6), suggesting minimal risk of bias due to missing data. Besides, to evaluate the influence of outliers, we winsorized continuous variables at the 1st and 99th percentiles and excluded individuals with TyGFI values beyond ± 3 SD. In CHARLS, this led to slight reductions in effect sizes for CVD (OR from 15.09 to 13.40) and stroke (OR from 21.12 to 17.62), but associations remained statistically significant with clear dose–response trends. In NHANES, estimates remained stable or even slightly increased, with consistent significance (Supplementary file 7–8). Overall, these analyses confirm that the associations between TyGFI and CVD/stroke risk are robust, not driven by selection bias or extreme values.

## Discussion

This investigation elucidates a robust and independent relationship between the combined TyG and FI indices and the risk of both CVD and stroke. Utilizing two large, nationally representative cohorts—CHARLS and NHANES—our analysis demonstrates that elevated TyGFI levels are consistently associated with increased odds of adverse cardiovascular outcomes. Furthermore, this association exhibits a clear dose–response pattern, as participants within the highest TyGFI quartiles experienced significantly greater risk elevations. Importantly, these findings remained stable after rigorous adjustment for a wide array of demographic, clinical, and lifestyle confounders. The observed associations were further corroborated through subgroup analyses, which revealed consistent effects across diverse demographic and clinical strata. Collectively, these results emphasize the potential clinical relevance of the TyGFI as an integrative risk marker for cardiovascular disease prevention and management. The association between higher TyGFI levels and increased risks of CVD and stroke likely reflects the combined effects of metabolic dysfunction and frailty on vascular health. Individuals in higher TyGFI quartiles had worse metabolic profiles, including higher BMI, glucose, HbA1c, and unfavorable lipid levels—all known risk factors for atherosclerosis. The clear dose–response pattern in both CHARLS and NHANES supports TyGFI as a reliable tool for cardiovascular risk stratification, even after adjusting for various confounders. Subgroup analyses further show that TyGFI can amplify risk in the presence of traditional factors like hypertension, diabetes, and dyslipidemia. The consistent findings across two large and diverse cohorts highlight TyGFI's broad applicability and potential clinical value.

Extensive research has established the TyG as a reliable surrogate marker for insulin resistance and a significant predictor of adverse cardiovascular and cerebrovascular outcomes. Prior studies have consistently demonstrated that elevated TyG levels are associated with increased risks of ASCVD, stroke, and mortality [9, 31–34]. Ding et al. (2021) confirmed that individuals with higher TyG levels were more likely to develop cardiovascular events, even in the absence of baseline CVD [35]. Cui et al. further highlighted the prognostic value of TyG, particularly in populations with compromised renal function [36]. In addition, Chen et al. reported a strong association between TyG and both all-cause and cardiovascular mortality [33]. In the domain of stroke research, elevated TyG has also been linked to increased incidence, recurrence, and poor prognosis. Yang et al. identified a significant association between higher TyG levels and the risk of ischemic stroke and its recurrence [37], while Cai et al. demonstrated that TyG was independently associated with in-hospital and ICU mortality in patients with critical stroke [31]. Moreover, longitudinal analyses conducted by Wu et al. and Huang et al. emphasized that persistent or increasing TyG trajectories over time were significantly correlated with elevated stroke risk [17, 38, 39]. These findings underscore the importance of TyG as a long-term indicator of metabolic risk. Emerging evidence has also begun to clarify the potential mechanisms behind these associations. Huo et al. showed that TyG mediated a substantial proportion of the relationship between body mass index and stroke, suggesting its involvement in key pathophysiological pathways linking obesity to vascular injury [19].

Mechanistically, the TyGFI index reflects a synergistic interplay between metabolic dysfunction and systemic physiological decline, both of which are central drivers of cardiometabolic vulnerability. The TyG index, a validated surrogate marker of insulin resistance, is associated with chronic low-grade inflammation, vascular endothelial dysfunction, and mitochondrial impairment. These pathological processes are likely mediated through the activation of nuclear factor kappa-light-chain-enhancer of activated B cells (NF-κB) and Janus kinase/signal transducer and activator of transcription (JAK/STAT) signaling pathways, which induce the expression of pro-inflammatory cytokines such as tumor necrosis factor-alpha (TNF-α), interleukin-6 (IL-6), and C-reactive protein (CRP). Simultaneously, insulin resistance downregulates key mitochondrial regulators including peroxisome proliferator-activated receptor gamma coactivator 1-alpha (PGC-1α) and sirtuin 1 (SIRT1), leading to impaired oxidative phosphorylation and cellular energy homeostasis [40–43]. Simultaneously, the FI has been mechanistically linked to dysfunction of cluster of differentiation 4–positive (CD4⁺) T cells, reduced levels of insulin-like growth factor 1 (IGF-1) and dehydroepiandrosterone sulfate (DHEA-S), as well as the accumulation of mitochondrial DNA (mtDNA) damage—all of which contribute to diminished physiological reserve and impaired stress adaptation capacity [44–47]. Moreover, emerging evidence underscores vascular endothelial dysfunction—defined by impaired nitric oxide (NO) bioavailability, increased arterial stiffness, and premature vascular aging—as a convergent pathological mechanism underlying both insulin resistance and frailty [48]. This mechanistic overlap provides a strong biological rationale for the multiplicative TyG × FI interaction: insulin resistance may amplify the vascular and inflammatory vulnerabilities conferred by frailty, while frailty may, in turn, exacerbate the systemic effects of metabolic stress. Meanwhile, recent findings by He et al. demonstrated that progression in frailty status significantly increased the risk of cardiovascular events, independent of traditional metabolic risk factors [17]. However, few investigations have attempted to combine metabolic and functional indicators into a single index.

Overall, this study exhibits multiple methodological and conceptual strengths that reinforce the robustness, generalizability, and clinical relevance of its findings. Notably, the utilization of two nationally representative and demographically distinct cohorts—CHARLS from China and NHANES from the United States—ensures broad external validity across populations with diverse sociocultural, genetic, and healthcare backgrounds. The consistency of results across these cohorts enhances the credibility of the observed associations and addresses an important limitation in prior cardiovascular research [34, 36, 49, 50].

Moreover, the study advances the field by proposing a novel composite risk indicator—TyGFI—that integrates the well-validated TyG, a surrogate marker of insulin resistance, with FI, a widely accepted measure of cumulative physiological deficits. While both TyG and FI have been independently associated with cardiovascular and cerebrovascular outcomes [17, 51, 52], their combination into a single metric represents a conceptual innovation. This dual-domain approach captures both metabolic and functional deterioration, enabling a more holistic and sensitive assessment of cardiovascular risk, particularly among aging individuals. In addition, the analytical framework is characterized by rigorous adjustment for a comprehensive set of covariates, including demographic, socioeconomic, behavioral, and clinical factors. The application of multivariable logistic regression, restricted cubic spline modeling, and stratified subgroup analyses ensures statistical robustness and allows for the detection of both linear and nonlinear relationships. Besides, the study also contributes to the literature by empirically demonstrating the added predictive value of TyGFI over its individual components. In doing so, it addresses a critical gap identified in previous studies that evaluated isolated metabolic indicators, such as TyG-BMI and TyG-WHtR, without accounting for functional health status [19, 53]. The integrative nature of TyGFI offers a more refined stratification tool for identifying individuals at elevated risk of cardiovascular and cerebrovascular events. Therefore, these strengths underscore the originality and applicability of TyGFI as a multidimensional risk marker, offering significant potential for incorporation into precision prevention strategies for cardiovascular and cerebrovascular disease.

However, despite the methodological rigor and cross-cohort validation, several limitations of this study should be acknowledged, particularly in the context of temporal dynamics and evolving risk factors. First, the analysis was based on baseline measurements of the TyG and FI, which are both time-sensitive indicators. However, growing evidence emphasizes the importance of longitudinal changes and cumulative exposure to these markers. For instance, Wu et al. and Huang et al. demonstrated that persistent elevation or upward trajectories in TyG over time were significantly associated with increased stroke risk in middle-aged and older adults [38, 39]. Similarly, He et al. showed that progression in frailty status over time was closely linked with elevated CVD incidence [17]. Second, although the study employed multivariable adjustment to control for confounding factors, its observational nature precludes definitive causal inference. While the associations identified are robust and consistent across two nationally representative cohorts, unmeasured confounding remains a possibility. Incorporating analytical approaches such as Mendelian randomization or instrumental variable analysis could strengthen causal interpretations, as illustrated by Jiang et al. [54]. Third, although CHARLS and NHANES represent different sociocultural and healthcare contexts, generalizability beyond Chinese and U.S. populations may be limited. Diverse populations with varying genetic backgrounds, dietary habits, and healthcare access may present distinct cardiometabolic trajectories [55–60]. Furthermore, different versions of the FI were applied in CHARLS and NHANES, which may introduce measurement variability and limit cross-cohort comparability. Nonetheless, although our primary aim was not to compare absolute frailty levels between different populations, we acknowledge that this heterogeneity may affect the broader generalizability of our findings. To strengthen external validity, future research should prioritize the use of harmonized frailty assessment protocols and consider pooled individual-level data to further validate and refine the TyGFI framework across diverse populations. Lastly, while our findings support the predictive value of the TyGFI as a composite indicator, its integration into clinical practice requires further validation. The multiplicative formulation (TyG × FI) is based on the hypothesis that metabolic and functional impairments interact synergistically to increase cardiovascular risk beyond additive effects. Although supported by epidemiological evidence, this approach remains exploratory. To advance the clinical implementation of the TyGFI index, two complementary methodological directions warrant further exploration. On one hand, modeling the longitudinal trajectories of TyGFI may offer nuanced insights into the temporal dynamics of cardiometabolic risk, thereby facilitating earlier identification of subclinical deterioration and enabling more timely evaluation of therapeutic responses. On the other hand, comparative analyses with additive or weighted composite indices are necessary to determine whether the multiplicative structure of TyGFI delivers superior prognostic utility in real-world settings. In particular, benchmarking TyGFI against widely used cardiovascular risk prediction models—such as the Framingham Risk Score and the ASCVD Risk Calculator—is an important next step to establish its relative performance and clinical relevance. Although such direct comparisons were beyond the scope of the present study, we recognize this as a key limitation and are planning follow-up analyses to evaluate the incremental value of TyGFI relative to these established tools. While the current study does not define prespecified clinical cut-off values, the consistently graded associations observed across TyGFI quartiles provide a practical basis for provisional risk stratification. Building upon this gradient, subsequent studies should employ bootstrap-based receiver operating characteristic (ROC) analyses to derive clinically meaningful thresholds. Furthermore, the added value of TyGFI in risk prediction models should be systematically evaluated through net reclassification improvement (NRI) and decision curve analysis (DCA), both of which quantify improvements in model discrimination and clinical benefit.

In summary, despite certain limitations, this study highlights the TyGFI as a novel and clinically accessible composite marker that integrates metabolic dysfunction and frailty. It demonstrates strong potential for improving cardiovascular and stroke risk stratification, particularly in aging populations.

## Conclusion

This study identified a strong and independent association between higher TyGFI levels and increased risks of cardiovascular disease and stroke in both CHARLS and NHANES cohorts. Participants with elevated TyGFI showed worse metabolic profiles and a higher burden of chronic conditions. The associations were dose-dependent and remained robust after adjusting for multiple confounders. Subgroup and spline analyses confirmed the consistency and nonlinearity of these relationships. These findings suggest that TyGFI is a practical and integrative marker for identifying individuals at higher cardiometabolic risk, especially in aging populations.

## Supplementary Information

Below is the link to the electronic supplementary material.

Supplementary Material 1.

## Data Availability

Data supporting the findings of this study are publicly available from the official websites of the CHARLS (http://charls.pku.edu.cn) and the NHANES (https://www.cdc.gov/nchs/nhanes/index.htm). Access to both datasets is granted for research purposes upon request or registration, in accordance with their respective data use policies.
